# Cost-utility analysis of esketamine and electroconvulsive therapy in adults with treatment-resistant depression

**DOI:** 10.1186/s12888-021-03601-8

**Published:** 2021-12-07

**Authors:** Kinza Degerlund Maldi, Peter Asellus, Anna Myléus, Fredrik Norström

**Affiliations:** 1 Department of Epidemiology and Global Health, Umeå University, SE-901 87 Umeå, Sweden; 2 Department of Clinical Sciences, Division of Psychiatry, Umeå University, Umeå, Sweden; 3 Department of Public Health and Clinical Medicine, Family Medicine, Umeå University, Umeå, Sweden

**Keywords:** Electroconvulsive therapy, Esketamine, Treatment-resistant depression, Cost-effectiveness, Markov model, QALY, ICER

## Abstract

**Background:**

Electroconvulsive therapy (ECT) has long been used for treating individuals with treatment-resistant depression (TRD). Esketamine has recently emerged as a new treatment for TRD due to its rapid antidepressant effects. To further inform the decision regarding choice of treatment, this paper aims to evaluate whether ECT or esketamine is the more cost-effective option.

**Methods:**

The cost-effectiveness was derived as cost per quality-adjusted life-year (QALY) using a Markov model from a societal and life-time perspective. The incremental cost-effectiveness ratio (ICER) was calculated. Health states included different depression and remission states and death. Data to populate the model was derived from randomised controlled trials and other research. Various sensitivity analyses were carried out to test the robustness of the model.

**Results:**

The base case scenario shows that ECT is cost-effective compared to esketamine and yields more QALYs at a lower cost. The sensitivity analysis shows that ECT is cost-effective in all scenarios and ECT dominates esketamine in 12 scenarios.

**Conclusions:**

This study found that, from a cost-effectiveness point of view, ECT should be the first-hand option for individuals with TRD, when other first line treatments have failed. Considering the lack of economic evaluation of ECT and esketamine, this study is of great value to decision makers.

## Introduction

Major depressive disorder is one of the most prevalent and debilitating forms of mental illnesses and a major cause of morbidity worldwide. In the United Kingdom (UK), it is estimated that 6.4% of the population suffer from the condition [1]. Major depressive disorder is usually treated with antidepressant medication (AD), but some individuals do not respond to treatment and they are considered suffering from treatment-resistant depression (TRD). There is no universally accepted definition of TRD, although the literature has commonly defined the condition as failure to respond to at least two trials of first line AD of both adequate duration and dose [2]. It is estimated that between 10 and 30% of individuals with major depressive disorder also have TRD with a significant impact on their daily life and productivity [2–5]. The societal impact of TRD is great due to its prevalence in the population, its impact on functioning, productivity and quality-of-life and its contribution to premature mortality [6].

Individuals with TRD can be treated with a combination of AD, mood stabilising medication, antipsychotic medication, psychological therapy and electroconvulsive therapy (ECT). ECT is often used as a “last option” when all other treatments have failed or not been tolerated. The treatment is not routinely considered for people with moderate depression unless their depression has not responded to multiple drug and psychological treatments [3]. ECT is considered highly effective in treating TRD, with remission rates between 50 and 70% [7]. Moreover, in a recent large observational study of older patients, ECT was associated with a lower one-year all-cause mortality and reduced suicide rates during three months after treatment [8]. Despite the consistent evidence that ECT is an effective treatment for patients with TRD, research suggest that it is underutilised [9–11]. This is partly due to limited availability of ECT, patient choice, stigma and a dated perception of the treatment [9–11]. It is also due to the fact that maintenance ECT (M-ECT) might be required, a practice that is uncommon in England [12] and not supported by UK National Institute for Health and Care Excellence (NICE) as routine treatment [13].

There has, however, been recent developments in treatments for TRD. The N-methyl-D-aspartate receptor antagonist ketamine, commonly used as a tranquiliser and pain medication, has emerged as a new treatment due to its rapid and robust antidepressant effects [14]. According to a systematic review [15], 77% of the included studies reported significant improvement in depressive symptoms among patients receiving ketamine or esketamine compared to the control group. The review concluded that ketamine is an effective treatment option for patients with major depressive disorder when administered via intravenous, intranasal and oral routes. The review included two studies where ketamine was administered intranasal with improvement in both studies. Both the Food and Drug administration (FDA) and the European Commission have approved intranasal esketamine in combination with a selective serotonin reuptake inhibitor (SSRI) or serotonin and norepinephrine reuptake inhibitor (SNRI) for adults with TRD [16, 17].

In an individual with TRD, ECT and esketamine are both viable treatment options. To further inform the decision regarding choice of treatment, an economic evaluation may be utilised as it compares the difference in cost and the difference in benefits between the options. In the case of an option being dominant, costing less and generating greater benefits than the alternative, it is unequivocally cost-effective. However, if an intervention generates more health to a higher cost than the option, it can still be cost-effective. In these situations, the incremental cost-effectiveness ratio (ICER) is calculated which demonstrates the additional cost per extra unit of health [18]. To our knowledge there is no cost-effectiveness analysis evaluating whether ECT or esketamine is the more cost-effective option.

### Aim

The aim of this paper is to evaluate whether ECT or esketamine is the more cost-effective option for treating TRD.

## Method

To determine which option is cost-effective, the ICER is estimated by calculating the difference in cost between ECT and esketamine divided by the difference in quality-adjusted life years (QALYs) between ECT and esketamine. The ICER is subsequently compared to a threshold value which will determine if the intervention is cost-effective [18]. The analysis is done in the context of the UK, and NICE recommends a threshold in the range of £20,000 to £30,000 per QALY gained [19]. However, NICE does not reject nor approve based on cost-effectiveness alone [20].

### The model

The cost-effectiveness analysis is based on a Markov model which defines the health and treatment states and possible consequences of the interventions. The prognosis of individuals is modelled, based on a set of possible transitions between these states over a series of discrete time periods. Cycles of 30 days was used in our Markov model as the cycle length is short enough to simulate the frequency of clinical events and treatment interventions and longer cycles can introduce more bias [21]. This model structure is more flexible than other model alternatives, such as decision trees, and allows for easy incorporation of relapses and recurrences [22]. This study is targeting a population with TRD where previous treatments have resulted in a lack of improvement and the next step in treatment would be either ECT or esketamine. It is therefore assumed that individuals enter the model at 45 years of age and the model lasts for 35 years, that is until the individuals are 80 years.

The Markov model requires three types of data: transition probabilities, health utilities and cost [23]. Data for the model was gathered from various sources, see Table 1 for the input parameters used in the analyses.

**Table 1:** Input parameters for the model. Costs are displayed in British pounds (GBP) 2019

	ECT	Source	Esketamine	Source
Transition probability				
Depression to remission (tpA2B)^a^ and (tpC2B)	0.696	(28)	0.392	(27)
Remission to depression (tpB2C)	0.108	(28)	0.0724	(26)
Remission to depression (tpB2C) for maintenance ECT	0.0300	(29)		
Transition probability for both ECT and esketamine				
Depression to death (tpA2G), (tpC2G) and (tpD2G)	0.00304	(31, 32)		
Depression to remission (tpD2E)	0.107	(30)		
Remission to depression (tpE2D)	0.232	(30)		
Remission to death (tpB2G and tpE2G) both ECT and esketamine				
45–49	0.000180	Lifetable		
50–54	0.000265	Lifetable		
55–59	0.000407	Lifetable		
60–64	0.000646	Lifetable		
65–69	0.00100	Lifetable		
70–74	0.00163	Lifetable		
75–79	0.00285	Lifetable		
Utility per cycle				
Remission	0.07	(33)		
Depression	0.05	(33)		
Cost per state and cycle age < 65 (age ≥ 65)				
State A and C	7932 (6321)		7210 (5599)	
State B, standard treatment after ECT	701 (118)		–	
State B, month 1 M-ECT and esketamine	3313 (2730)		2882 (2299)	
State B, month 2 and following months M-ECT and month 2–5 esketamine	2161 (1578)		1583 (1000)	
State B, month 6 esketamine	–		1890 (1307)	
State D	2729 (1119)		2729 (1119)	
State E and F	701 (118)		701 (118)	

^a^ tp = transition probability, that is, the probability of the individual moving from one state to the other. tpA2B indicates the probability of the individual transitioning from state A (depression) to state B (remission)

**Fig. 1:**
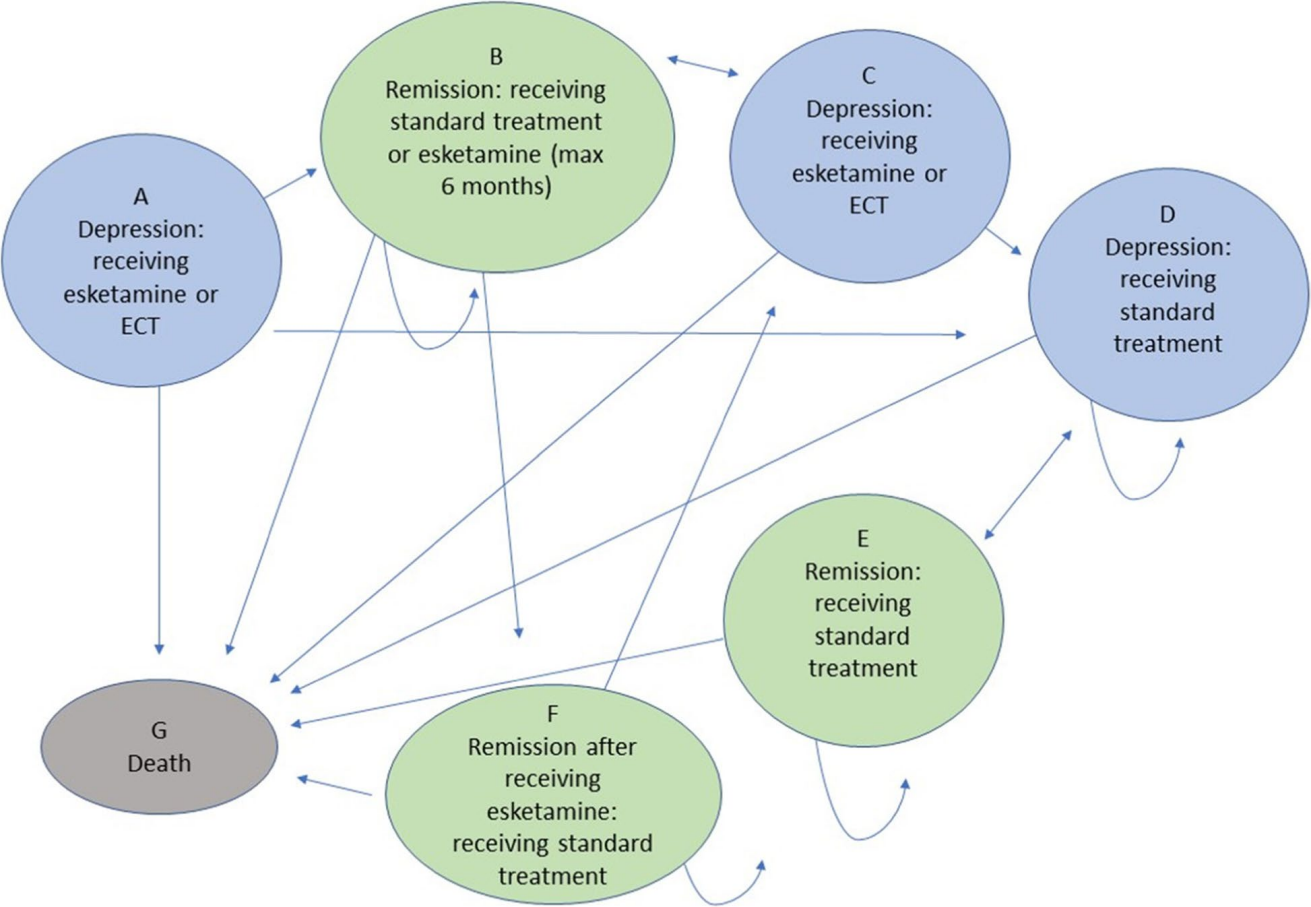
Markov model schematic with the states and transition patterns. All individuals started in state A as depressed and received either electroconvulsive therapy (ECT) or esketamine. The possible transitions between the states are illustrated with arrows. Blue states indicate ongoing depression and green states indicate remission

The Markov model was constructed in Excel and an overview with arrows indicating possible transition patterns can be found in Fig. 1. Individuals started in “state A depression” and received either esketamine or ECT. Individuals who remitted on esketamine transitioned to “state B remission” and received esketamine for a maximum of six months. They transitioned to “state F remission” after six months, given that they were still in remission, and received standard treatment. If the individual relapsed while receiving esketamine in “state B remission”, they transitioned to “state C depression” and received esketamine at a higher dose. The transition pattern from esketamine was based on the treatment protocol by Janssen [24].

Individuals who remitted on ECT transitioned to “state B remission” and received standard treatment. If the individual relapsed, they transitioned to “state C depression” and received ECT. Individuals who did not remit on ECT or esketamine transitioned to “state D depression” and in the case of treatment only received standard treatment. Individuals who remitted in “state D depression” transitioned to “state E remission”.

An additional model was constructed where individuals received M-ECT. In this model individuals who remitted on ECT and transitioned to “state B remission” received M-ECT. If they relapse during M-ECT they transitioned to “state C depression” and received ECT.

### Interventions

#### Standard treatment

Individuals received AD in all states and visited their general practitioner (GP) once a month for this [25].

#### Esketamine

The treatment regimen was based on the treatment protocol by Janssen [24]. In depression (state A and C) the treatment consisted of two doses a week at a community mental health centre (CMHT). The starting dose was 56 mg, followed by a dose of 56 mg or 84 mg [24]. Esketamine was prescribed by a psychiatrist and administered by a nurse. Individuals continued to receive esketamine from a nurse at the CMHT, but at a lower frequency, in remission. The maintenance dose of esketamine was recommended at 56 or 84 mg once a week the first four weeks followed by a further reduction to one dose every other week, for a maximum of six months. According to the literature, around half of the individuals received the higher dose and half the lower [26, 27], thus, an average dose was used. For example, for the first four weeks the total dose for an individual receiving 56 mg was 448 mg and the total dose for an individual receiving 84 mg was 644 mg, resulting in an average dose of 546 mg. The individual had two psychiatrist appointments, one in the beginning of the cycle to be prescribed esketamine and one in the end for follow-up.

#### ECT

According to NICE, ECT is usually given twice a week, thus in depression (state A and C) the individual had eight sessions of ECT and two psychiatrist appointment, one in the beginning of the cycle to be prescribed ECT and one at the end of the cycle for follow-up [13]. The model was based on one cycle of ECT in the depression state and standard treatment in remission. In the main model, ECT is not given as maintenance treatment as this appears to be fairly uncommon in the UK, with about 9.6% of individuals receiving ECT continuing with M-ECT [12]. Moreover, NICE reports little evidence regarding the value of M-ECT. However, to make the study more applicable to countries where M-ECT is used, a maintenance model with M-ECT was constructed. Individuals who remitted on ECT continued to receive M-ECT weekly for six weeks followed by twice a month.

### Transition probabilities

The Markov model was populated with transition probabilities derived from various studies and converted to monthly probabilities by using eq. 1 and 2.

1 $$r=-\frac{\left[\ln \left(1-P\right)\right]}{t}$$

2 $$p=1-{\exp}\left\{- rt\right\}$$

Where *r* = rate, *p* = probability, *t* = time period of interest.

The transition probability of remitting on esketamine was derived from a randomised, double-blind, active-controlled study where 342 patients from Europe, North America and Brazil were randomised to either placebo or twice a week intranasal esketamine 56 or 84 mg plus an AD [27]. Remission rates in this study were 36.0% for the 56 mg group and 38.8% for the 84 mg group during four weeks.

The transition probability of remitting on ECT and relapsing while receiving AD after successful ECT was derived from a randomised controlled trial. Out of 206 eligible patients from London UK, 46 entered the study of which 22 received ECT. Remitters were followed up for six month to capture relapse [28]. Remission from ECT was 59.1% during the study and relapse after six months was 50%. For the maintenance model with M-ECT, the transition probability of relapsing while receiving M-ECT was derived from a Swedish randomised controlled trial. Out of 200 eligible patients from four different hospitals in Sweden, 56 were randomised to either receive M-ECT and pharmacotherapy or pharmacotherapy [29]. During the one year follow up, 31% of the medication resistant individuals receiving M-ECT experienced a relapse.

The transition probability of relapsing while receiving esketamine maintenance treatment was derived from a double-blind, randomised clinical trial evaluating the long-term use of esketamine [26]. Out of 1097 eligible patients from Europe, North America and Brazil, 297 were randomised to either receive esketamine or placebo. During the 17.7 weeks maintenance phase 26.7% individuals experienced a relapse.

The maintenance phase of esketamine was maximum six months and individuals transitioned to standard treatment after six months [24]. The transition probability of moving to this state was set as the same probability of remaining in remission. The transition probability of relapsing was the same as relapsing on standard treatment.

The transition probability of remitting from standard treatment and relapsing from standard treatment, was derived from the STAR*D trial. This is the largest open-label, pragmatic trial that has been undertaken to examine the treatment of major depressive disorder [30]. In this trial, 3671 American individuals received AD medication, which was augmented and changed in those without sufficient response. The transition probability was derived from the 390 individuals who had not benefited sufficiently from two different medication trials, alike the definition of TRD. The remission rate in this study was 13.7% during 5.6 weeks and the rate of relapse was 64.6% during 3.1 months.

The transition probability of death while suffering from depression was derived from two studies. A study using a Swedish register of 118,774 individuals concluded that the increased risk of death among individuals with TRD is 1.35 times higher compared to depression [31]. This increased risk was applied to a prospective study of 20,320 individuals which estimated mortality in people with depression in the UK [32]. The study concluded that 11.6% of the study population died from all-causes over an average follow up time of 4.5 years. The transition probability of death while in remission was derived from a lifetable and calculated over five-year intervals, 45–49 years, 50–54 years and so on.

### Utility scores

QALYs aim at capturing the health of a specific state. One year of perfect health is valued as 1, and death is valued at 0. Utility scores were set as 0.81 QALYs for remission and 0.57 QALYs for depression, and converted to monthy utility scores. The utility scores originates from a longitudinal study using the validated EQ-5D questionnaire to investigate the quality of life in individuals with depression in Sweden [33]. The utility scores have been used in at least two studies similar to ours [34, 35].

### Costing

The study adopts a societal perspective by including direct costs related to the utilisation of medical resources, such as healthcare visits, and indirect costs related to the loss of productivity and informal care [36–38]. In contrast, a healthcare perspective only includes the direct costs. It has been argued that the loss of productivity is especially important in mood disorders as it accounts for a large proportion of the total cost burden. Individuals suffering from mood disorders lose, on average, more work days compared to individuals suffering from other chronic conditions [39].

If wages are used to quantify productivity loss, health will be valued higher for high-income earners and men, over that of low-income earners and women. For equity reasons the proposed solution is to apply a general wage rate, that is, the average wage of the country and payroll tax [39]. Therefore, the average full-time wage of £2340, part-time wage of £680 and payroll tax of 12% (applied to income between £702.01 and £3863 per month) were used for this study [40]. Calculations were performed under the assumption of employment until age 65 years, which is the general retirement age in the UK. After retirement (≥65 years of age) no productivity loss was assumed. When necessary, costs were converted to 2019 years value pound sterling (GBP) by the Bank of England inflation calculator. Cost for each state is displayed in Table 1.

#### Direct costs

Most of the costs for TRD were taken from Table 2 in a study by McCrone et al. [25], apart from the cost for GP, psychiatrist and nurse visits, which was taken from another source [41]. The cost for ECT was derived from NICE which has estimated one session to cost £558 [13] and the cost for esketamine was derived from Jansen at £163/28 mg [24].

**Table 2:** Cost-effectiveness of ECT and esketamine *main model* - base case and sensitivity analysis

	ECT		Esketamine		Incremental		ICER
	Cost	QALY	Cost	QALY	Cost	QALY	
Base case							
Societal perspective	453,693	14.85	456,211	14.26	−2517	0.59	ECT dominates^a^
Healthcare perspective	124,530	14.85	120,390	14.26	4140	0.59	6969
Sensitivity analysis							
(1a) Doubling the productivity loss	681,736	14.85	688,301	14.26	−6565	0.59	ECT dominates
(1b) Halving the productivity loss	339,673	14.85	340,165	14.26	−493	0.59	ECT dominates
(2a) ECT six times per cycle – societal	450,071	14.85	456,211	14.26	−6140	0.59	ECT dominates
(2b) ECT six times per cycle – healthcare	121,026	14.85	120,390	14.26	636	0.59	1070
(2c) ECT 12 times per cycle – societal	460,939	14.85	456,211	14.26	4728	0.59	7959
(2d) ECT 12 times per cycle – healthcare	131,539	14.85	120,390	14.26	11,149	0.59	18,768
(3a) Increasing the remission rate from esketamine to 0.5 – societal	453,694	14.85	458,449	14.36	−4755	0.49	ECT dominates
(3b) Increasing the remission rate from esketamine to 0.5 – healthcare	124,530	14.85	123,583	14.36	947	0.49	1927
(4a) Decreasing the remission rate from ECT to 0.5 – societal	453,364	14.42	456,211	14.26	−2847	0.16	ECT dominates
(4b) Decreasing the remission rate from ECT to 0.5 – healthcare	119,350	14.42	120,390	14.26	−1040	0.16	ECT dominates
(5a) Increasing the remission rate the 2nd time the individuals received ECT/esketamine 0.9 – societal	448,644	16.15	472,803	15.08	−24,159	1.08	ECT dominates
(5b) Increasing the remission rate the 2nd time the individuals received ECT/esketamine 0.9 – healthcare	141,026	16.15	148,069	15.08	−7044	1.08	ECT dominates
(6a) Five-year time horizon – societal	105,078	3.00	116,086	2.81	−11,008	0.19	ECT dominates
(6b) Five-year time horizon – healthcare	31,691	3.00	31,284	2.81	406	0.19	2086
(7a) Lowering QALYs (0.3) for ECT treatment during depression and increasing QALYs (0.85) for remission after esketamine and AD – societal	453,694	15.04	456,211	14.54	−2517	0.5	ECT dominates
(7b) Lowering QALYs (0.3) for ECT treatment during depression and increasing QALYs (0.85) for remission after esketamine and AD – healthcare	124,530	15.04	120,390	14.54	4140	0.5	8296
(8a) Esketamine was given as long as the individual was in remission – societal	453,694	14.85	458,749	14.41	−5055	0.45	ECT dominates
(8b) Esketamine was given as long as the individual was in remission – healthcare	124,530	14.85	124,358	14.41	172	0.45	387
(9a) No discounting applied – societal	466,497	15.22	469,603	14.60	−3106	0.61	ECT dominates
(9b) No discounting applied – healthcare	127,589	15.22	123,191	14.60	4399	0.61	7156

^a^ Dominates = more QALY at a lower cost

Costs are displayed in British pounds (GBP) 2019

#### Indirect costs

Loss of productivity was weighted to account for 54% of the population being unemployed and 13% working part-time [25] while in the depression state and for 23% [23, 25] of the individuals being unemployed in the remission state. These figures have been used elsewhere in a similar study [23]. The usage of informal care was derived from McCrone et al. [25] and the cost from the Office of National Statistics by applying the mean hourly wage [40]. Lastly, individuals travel to the CMHT appointment was assumed to be the similar to the London daily cap of £18.80 even if individuals are outside of London [42]. Yearly discounting of 3.5% was applied to both cost and health.

### Sensitivity analysis

Sensitivity analyses were performed by alternating one key parameter at a time in the model. The study has adopted a societal perspective, but where applicable, the ICER from a healthcare perspective was also estimated.

1. Doubling and halving productivity loss.
2. Changing the frequency of ECT to six and 12 times per cycle.
3. Increasing the remission rate from esketamine to 0.5.
4. Decreasing the remission rate from ECT to 0.5.
5. Increasing the remission rate the second time individuals received ECT/esketamine in the depression state to 0.9.
6. Applying a five-year time horizon.
7. Lowering QALYs to 0.3 during ECT treatment while depressed and increasing QALYs to 0.85 for remission after esketamine and after AD.
8. Esketamine was given as long as the individual was in remission, that is, longer that six months.
9. Applying no discounting for both costs and utility.

## Results

Table 2 presents the results from the main model base case and the sensitivity analysis. The results show that from a societal perspective ECT dominates esketamine, that is, ECT generates more QALYs at a lower cost. From a healthcare perspective, the ICER, comparing ECT to esketamine is £6969 per QALY gained, i.e. ECT is more efficient and costlier. The ICER is below the lower border of the suggested threshold from NICE. The sensitivity analyses confirm these findings. In all scenarios ECT appears cost-effective compared to esketamine and in 12 scenarios ECT dominates esketamine, see Table 2.

Table 3 presents the results from the maintenance model with M-ECT. The base case from a societal perspective indicates that ECT followed by M-ECT is cost-effective compared with esketamine. The ICER is £27,070 per QALY gained. From a healthcare perspective the ICER is £38,922 and is thus above the suggested threshold from NICE ranging from £20,000 to £30,000 per QALY gained. The sensitivity analyses of the maintenance model indicates that ECT followed by M-ECT is cost-effective from a societal perspective in all sensitivity analyses but three, see Table 3. Firstly, the ICER from sensitivity analysis 1b, halving the productivity loss, is £31,138 which is above the threshold. Secondly, the ICER from sensitivity analysis 5, where the remission rate is increased the second time the individual received ECT or esketamine, is £32,059 which is above the threshold. Thirdly, the ICER from sensitivity analysis 8, where the individual received esketamine as long as they were in remission, was £41,660 also above the threshold. From a healthcare perspective the estimated ICERs of the maintenance model were above the threshold in all analysis.

**Table 3:** Cost-effectiveness of ECT and esketamine *maintenance model* with M-ECT - base case and sensitivity analysis

	ECT		Esketamine		Incremental		ICER
	Cost	QALY	Cost	QALY	Cost	QALY	
Base case							
Societal perspective	525,707	16.83	456,219	14.26	69,488	2.57	27,070
Healthcare perspective	220,303	16.83	120,392	14.26	99,911	2.57	38,922
Sensitivity analysis							
(1a) Doubling the productivity loss	736,918	16.83	688,314	14.26	48,604	2.57	18,935
(1b) Halving the productivity loss	420,102	16.83	340,172	14.26	79,930	2.57	31,138
(2a) ECT six times per cycle during depression – societal	522,167	16.83	456,219	14.26	65,948	2.57	25,691
(2b) ECT six times per cycle during depression – healthcare	216,879	16.83	120,392	14.26	96,487	2.57	37,588
(2c) ECT 12 times per cycle during depression – societal	532,787	16.83	456,219	14.26	76,568	2.57	29,828
(2d) ECT 12 times per cycle during depression – healthcare	227,152	16.83	120,392	14.26	106,760	2.57	41,590
(3a) Increasing the remission rate from esketamine to 0.5 – societal	525,707	16.83	458,462	14.36	67,245	2.46	27,290
(3b) Increasing the remission rate from esketamine to 0.5 – healthcare	220,303	16.83	123,587	14.36	96,717	2.46	39,250
(4a) Decreasing the remission rate from ECT to 0.5 – societal	490,004	15.54	456,219	14.26	33,784	1.28	26,326
(4b) Decreasing the remission rate from ECT to 0.5 – healthcare	164,091	15.54	120,392	14.26	43,699	1.28	34,052
(5a) Increasing the remission rate the 2nd time the individuals received ECT/esketamine 0.9 – societal	595,181	18.89	472,830	15.08	122,351	3.82	32,059
(5b) Increasing the remission rate the 2nd time the individuals received ECT/esketamine 0.9 – healthcare	332,551	18.89	148,080	15.08	184,471	3.82	48,336
(6a) Five-year time horizon – societal	127,153	3.21	116,088	2.81	11,066	0.4	27,570
(6b) Five-year time horizon – healthcare	65,956	3.21	31,285	2.81	34,671	0.4	86,383
(7a) Lowering QALYs (0.3) for ECT treatment during depression and increasing QALYs (0.85) for remission after esketamine and AD – societal	525,707	16.99	456,219	14.55	69,488	2.45	28,378
(7b) Lowering QALYs (0.3) for ECT treatment during depression and increasing QALYs (0.85) for remission after esketamine and AD – healthcare	220,303	16.99	120,392	14.55	99,911	2.45	40,802
(8a) Esketamine was given as long as the individual was in remission – societal	525,707	16.83	424,948	14.41	100,759	2.42	41,660
(8b) Esketamine was given as long as the individual was in remission – healthcare	220,303	16.83	124,358	14.41	95,945	2.42	39,670
(9a) No discounting applied – societal	540,562	17.22	469,612	14.60	70,950	2.42	27,063
(9b) No discounting applied – healthcare	227,209	17.22	123,193	14.60	104,016	2.42	39,676

Costs are displayed in British pounds (GBP) 2019

## Discussion

This is, to our knowledge, the first study that evaluates whether ECT or esketamine is to recommend from a cost-effectiveness perspective among individuals with TRD. The base case from the main model indicated that ECT is cost-effective compared with esketamine. The results show that the absolute cost of ECT is slightly lower than the cost for esketamine and ECT generates more QALYs. Results from the sensitivity analyses demonstrate the robustness of these findings. There were some changes to the ICER, but all the sensitivity analyses resulted in ECT being cost-effective compared with esketamine. The results from the maintenance model with M-ECT somewhat supports these finding.

A few estimates in the sensitivity analysis from the main model are particularly noteworthy. Analysis number 7 (lowering QALYs for ECT, increasing QALYs for remission with esketamine and AD) still generated more QALYs for ECT than esketamine. This is due to the higher remission from ECT resulting in more QALYs despite reducing QALYs during ECT treatment. This is an important finding as studies have reported lower QALYs from ECT than from other treatments. QALYs from ECT treatment have been reported as low as 0.3 and 0.56 [22, 43] while QALYs in remission after AD has been reported as high as 0.85 [44, 45]. Nguyen and Gordon [46] have reported a higher disutility from ECT at 0.104 compared with AD at 0.066. Alike, research regarding QALYs in schizophrenia has suggested different utility scores depending on side effects from the medication [47]. For individuals experiencing difficult side effects or stigma from ECT [48], it is possible that the QALYs would differ between ECT and esketamine. Our sensitivity analysis from both models supports the fact that ECT would still be cost-effective.

Sensitivity analysis 6a (five-year time horizon) and 8a (esketamine in remission) are interesting findings. In the main model, ECT dominates esketamine in both scenarios, indicating that the time horizon which esketamine is administered does not change the cost-effectiveness. This in combination with sensitivity analysis number 5a and b (increasing the remission rate the second time the individual received esketamine to 0.9) suggests that administrating more esketamine, makes it less cost-effective. However, the maintenance model with M-ECT yields slightly different results. In this model the ICER falls above the threshold in sensitivity analysis 5 and 8.

The model included loss of productivity by accounting for unemployment and part-time work in the depression state and unemployment in the remission state, similarly to another cost-effectiveness analysis [23]. However, the loss of productivity did not include part-time employment in the remission state. As indicated by the sensitivity analysis, halving and doubling the productivity loss still results in ECT being cost-effective in the main model and the ICER falls just above the threshold in the maintenance model.

Other cost-effectiveness studies have assessed esketamine compared with AD and have concluded that esketamine is not cost-effective. Ross and Soeteman [49] compared esketamine to AD by using a decision-analytic model. They concluded that esketamine was likely not cost-effective and their sensitivity analysis did not find any realistic cost-effective scenarios. Agboola et al. [50] likewise evaluated the cost-effectiveness of esketamine plus background AD compared with AD alone in patients with TRD and reached similar conclusions. The current study is a valuable complement to these studies, as we now have evidence regarding ECT being cost-effective compared with esketamine.

ECT is cost-effective due to its higher remission rate, compared to esketamine, at a slightly lower cost in the main model. The remission rate for ECT was 69.6 and 50% in the sensitivity analysis while the study used for esketamine remission rate reported between 36 and 38.8%. Critique towards a relaxed inclusion of participants and a relaxed definition of TRD in the esketamine trials have been voiced [51]. The trials only required individuals to have two failed treatments of any two AD, enabling inclusion of patients in whom only SSRIs had failed. In the study by Fedgchin et al. [27] only 30.1 and 48.2% had three or more previous AD and in the study by Daly, et al. [26] 21.1 and 26.2% had tried more than two AD. Thus, clinicians might find that esketamine demonstrates less efficacy among real-world patients with higher levels of treatment resistance. The trial also included a new AD that the individual had not tried before, making it a possibility that part of the treatment effect came from the AD. The treatment protocol used for the maintenance phase has been evaluated and Nijs et al. [52], suggests that altering the dose of esketamine might optimise the treatment. The treatment protocol stipulates that during the maintenance phase individuals receive esketamine once a week following a further reduction to every other week. Researchers adjusted the dose for individuals relapsing during the reduction and saw that 47% improved by increasing the dose from bimonthly to weekly. These finding suggest that an individualised treatment plan of esketamine might optimise treatment response in the real world. However, with an increased dose comes an increased cost and potentially a higher risk for drug abuse.

The study by Eranti et al. [28] used for the transition probabilities for remission and relapse from ECT, only included 22 participants in the first phase and 16 participants in the follow-up phase. The most common reason for exclusion was not consenting to ECT and there was no statistically significant difference in mean age or sex ratio between the eligible patients who consented and those who declined to participate. Moreover, ECT was not administered for a fixed schedule, rather it was given until treatment response was evident but the mean duration was similar to the control group which was three weeks. This invites to some uncertainty regarding the transition probabilities. However, similar transition probabilities have been reported from another study [53], and the sensitivity analysis with a lower remission rate did not result in any noteworthy changes to the ICER. On the other hand, the strength of the study by Eranti et al. [28] and the study by Rush et al. [30], used for the relapse and remission rates for standard treatment, was that the same patients who remitted were followed-up for relapse, mimicking real-world conditions. Moreover, the studies used to populate the model had similar definitions of TRD. As mentioned previously, the esketamine studies had a more relaxed criteria, while the other studies applied the definition of two failed AD.

### Strengths and limitations

A strength of the study is that it is one of the first of its kind, estimating the cost-effectiveness of ECT and esketamine. The study adopts a societal perspective and has included a broad range of indirect costs of TRD, rather than the cost of depression. This might result in a more accurate estimate of the actual cost of TRD. The study used a UK context with regards to costs and how the health care system is organised. Nonetheless, the findings are generalisable to contexts with similar healthcare systems and the maintenance model is applicable to countries using M-ECT.

Several limitations need to be considered in the study. Firstly, the studies used for QALYs employed EQ-5D to generate these and it has been argued that EQ-5D might not be adequately sensitive to changes in functioning and quality of life due to mental illness [54]. Secondly, the study utilised to derive the remission and relapse rates for esketamine had a low threshold for TRD. Thirdly, the models did not include disutility for side-effect as the research on QALYs during esketamine treatment is limited. Fourthly, the study did not address heterogeneity of different population characteristics. Lastly, the lack of long-term research of esketamine is a limitation as it could make the estimates unreliable. However, the sensitivity analysis using a five-year time horizon did not yield noteworthy changes to the cost-effectiveness. Adding to this, randomised controlled trials were used to populate the model, which can be a limitation, as they might not reflect real-world conditions. Thus, further economic evaluations are warranted with longitudinal or naturalistic studies.

## Conclusion

This study found that, from a cost-effectiveness point of view, ECT should be the first-hand option for individuals with TRD, when other first line treatments have failed. The time horizon did not change the estimates noteworthy and there was no scenario in the main model where esketamine was cost-effective. Nonetheless, esketamine could potentially be an option for individuals not able to undergo ECT. Further research regarding potential subgroups of individuals where esketamine treatment could be cost-effective is warranted. Considering the lack of economic evaluation of ECT and esketamine, this study is of great value to decision makers.

## Data Availability

All data generated or analysed during this study are included in this published article.

## Abbreviations

AD: Antidepressant medication
CMHT: Community mental health center
ECT: Electroconvulsive therapy
FDA: Food and Drug Administration
ICER: Incremental cost-effectiveness ratio
QALY: Quality-adjusted life years
SNRI: Serotonin and norepinephrine reuptake inhibitor
SSRI: Selective serotonin reuptake inhibitor
TP: Transition probability
TRD: Treatment resistant depression
NICE: National Institute for Health and Care Excellence
UK: United Kingdom
